# A Novel *ESRRB* Deletion Is a Rare Cause of Autosomal Recessive Nonsyndromic Hearing Impairment among Pakistani Families

**DOI:** 10.4061/2011/368915

**Published:** 2011-09-25

**Authors:** Kwanghyuk Lee, Saadullah Khan, Muhammad Ansar, Regie Lyn P. Santos-Cortez, Wasim Ahmad, Suzanne M. Leal

**Affiliations:** ^1^Department of Molecular and Human Genetics, Baylor College of Medicine, Houston, Tx 77030, USA; ^2^Department of Biochemistry, Faculty of Biological Sciences, Quaid-I-Azam University, Islamabad 45320, Pakistan

## Abstract

Mutations in the estrogen-related receptor beta (*ESRRB*) gene is the underlying cause of autosomal recessive nonsyndromic hearing impairment (ARNSHI) due to the DFNB35 locus which maps to 14q24.3. A genome scan of a large consanguineous Pakistani pedigree with ARNSHI established linkage with a maximum multipoint LOD score of 4.2 to the 14q24 region and the region of homozygosity contained the *ESRRB* gene. Sequencing of the *ESRRB* gene using DNA samples from hearing-impaired family members uncovered a novel three-nucleotide deletion c.1018_1020delGAG (p.Glu340del). The deletion segregates with hearing impairment in the pedigree and was not observed in 500 control chromosomes. The deletion of glutamic acid residue occurs in the ligand-binding domain of ESRRB protein. It is expected that the deletion affects the ligand-binding activity of the domain in ESRRB, which leads to the ARNSHI.

## 1. Introduction

Hearing impairment (HI) has been associated with estrogen physiology for decades. Turner's syndrome in females and Noonan's syndrome in males are both marked by hypoestrinism and include the HI phenotype. Variations in hearing function have also been found according to gender, menstrual cycle or menopausal state, estrogen therapy, and oral contraceptive use, which implicate estrogen as a primary requirement for hearing preservation [1]. Recently, autosomal recessive nonsyndromic hearing impairment (ARNSHI) locus DFNB35 was shown to be due to mutations in the estrogen-related receptor beta or *ESRRB *gene (MIM 602167; 2). *ESRRB *belongs to a subfamily of orphan nuclear receptors that are structurally related to estrogen receptors (ER) but do not directly bind estrogen. The ESRR proteins have DNA-binding and ligand-binding domains, which are both required for transcriptional regulation of ER target genes. The ligand-binding domain (LBD) provides structural stability and influences the binding affinity of the ESRRB protein [2]. Here, we report a novel deletion mutation of glutamic acid residue in LBD domain of *ESRRB* gene which is responsible for ARNSHI. 

## 2. Material and Methods

**Figure 1:**
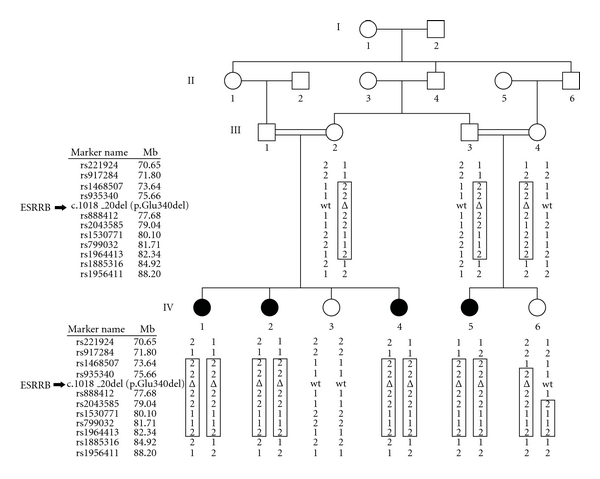
Pedigree drawing of family 4243. *Filled* symbols represent individuals with ARNSHI, and *clear* symbols represent hearing individuals. Displayed under each individual are the SNP markers within the region of the *ESRRB* gene and the p.Glu340del mutation. The haplotype segregating with the ARNSHI phenotype is shown within a *box*. The homozygous region that is found only in ARNSHI individuals is bounded by rs917284 (chr14 : 71.8 Mb) and rs2043585 (chr14 : 79.0 Mb). The black arrow indicates the position of *ESRRB* gene amid the SNP marker loci.

The study was approved by the Institutional Review Boards of the Quaid-I-Azam University and the Baylor College of Medicine and Affiliated Hospitals prior to initiation. Informed consent was obtained from all members from family 4243 who participated in the study. Family 4243 (Figure 1) is a consanguineous kindred from the Sairiki-speaking region of Punjab, Pakistan. The family segregates ARNSHI of prelingual onset and has no history of environmental exposure to factors that may cause HI, for example, infection, ototoxic medication, and trauma. Syndromic and vestibular features were ruled out through careful physical examination. To determine the severity of HI, air conduction testing was performed using a portable audiometer. 

Genomic DNA was extracted from venous blood which was provided by five hearing and four HI family members (Figure 1). The nine DNA samples from this family underwent a whole genome linkage scan at the Center for Inherited Disease Research (CIDR) using the Illumina Linkage Panel IV-b which contains 6,090 SNP markers. Data quality control was performed using PedCheck [3] to check for genotyping error which resulted in Mendelian inconsistencies, while MERLIN [4] was used to detect occurrence of double recombination events over short genetic distances, which are most likely due to genotyping error. Two-point linkage analysis was carried out using MLINK of the FASTLINK package [5], and multipoint linkage analysis was performed with Allegro [6], while haplotypes were reconstructed using SimWalk2 [7]. An autosomal recessive mode of inheritance with complete penetrance and a disease allele frequency of 0.001 were used in the analysis. Marker allele frequencies were estimated from observed and reconstructed founders from family 4243 and 73 additional families who underwent genome scan at the same time at CIDR. For the multipoint linkage analysis, genetic map positions were determined according to the Rutgers combined linkage-physical map of the human genome [8] using the human reference sequence (Build 36) to determine the physical map position, and then, interpolation was performed to place the markers on the Rutgers map.

Exon primers for *ESRRB* (RefSeq NM_004452.3) gene were designed using Primer3 software [9]. PCR-amplified products were purified with ExoSAP-IT (USB Corp., Cleveland, Ohio, USA) and sequenced with the BigDye Terminator v3.1 Cycle Sequencing Kit and the ABI 3730 DNA Analyzer (Applied Biosystems Inc, Foster City, Calif, USA). Sequencher software V4.9 (Gene Codes Corp., Ann Arborich, USA, M) was used to assemble and analyze DNA sequences.

## 3. Results and Discussion

**Figure 2:**
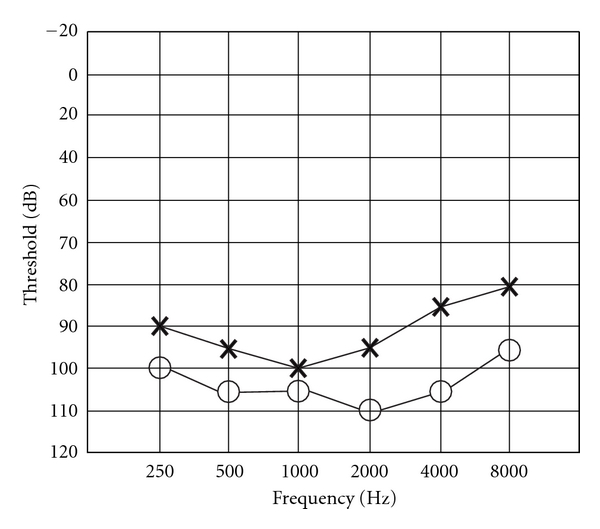
Audiogram of individual IV-4 demonstrates bilateral severe-to-profound HI at all frequencies. Air conduction threshold testing was performed for the right (*circles*) and left (*crosses*) ears.

The audiogram of HI individual IV-4 displays bilateral, severe-to-profound HI affecting all frequencies (Figure 2). This is consistent with the previous description of prelingual, bilateral profound hearing loss across all frequencies for *ESRRB-*related HI [2]. 

For family 4243, a maximum two-point LOD score of 3.04 (*θ* = 0) was observed at marker rs935340 (chr14:75.66 Mb; see Table 1). A significant maximum multipoint LOD score of 4.16 was obtained at two marker loci, rs935340 and rs888412 (77.68 Mb). The 3-unit support interval and the region of homozygosity that was observed only in HI individuals (Table 1; Figure 1) fall between markers rs917284 (71.81 Mb) and rs2043585 (79.04 Mb). The linkage region spans 10.08 cM within 14q24.2–q31.1 and contains 7.23 Mb of sequence. A total of 83 genes are found within the linkage interval, including the known ARNSHI gene, *ESRRB*. Since variants within the *ESRRB *gene are known to be involved in NSHI [2], the exon regions of *ESRRB* gene were sequenced. 

Sequencing of the *ESRRB *gene in family 4243 revealed a novel deletion c.1018_1020delGAG (p.Glu340del) in exon 8 which segregates with ARNSHI (Figure 1). The deletion was not annotated in the dbSNP database and 1000 Genomes project. Additionally, exon 8 of *ESRRB* was sequenced in 250 unrelated hearing individuals from Pakistan; the deletion was not found in 500 control chromosomes.

The glutamic acid residue at position 340 is the second residue within *α*-helix 8 at the LBD of the ESRRB protein. In order to form a hypothesis on the effect of its deletion to protein structure, ESRRB*-*like proteins from both human and nonhuman species were identified from the UniProt Knowledgebase [10] using blastp [11] and aligned via ClustalW [12]. The Glu340 residue is invariant for ESRRB and other steroid receptor proteins in 39 nonhuman species, including 7 mammalian, 1 avian, 2 reptilian, 3 amphibian, 17 fish, 2 ascidian, 3 mollusk, and 4 arthropod. It is also conserved in 32 human nuclear receptor proteins, which indicates that this residue is essential to the structure of the LBD. Using the SWISS-MODEL Workspace [13], the LBD of ESRRB was modeled after the LBD of human proteins ESRRG (PDB ID: 2GPO) [14] and PPARG (PDB ID: 3DZY) [15]. The Glu340 residue forms a hydrogen bond with Glu337, the last of three residues of the *α*-*α* corner between *α*-helices 7 and 8. This hydrogen bond marks the beginning of the formation of *α*-helix 8. Glu340 also forms two hydrogen bonds with Arg388, the first residue of *α*-helix 10. Removal of Glu340 dissolves these hydrogen bonds and also results in the rotation of the side chains of residues 336–339 along the helical axis. Residues at positions 336–340 do not have direct contact with ligand. However, the removal of hydrogen bonds due to p.Glu340del is expected to affect the stability of *α*-helix 8 and its conformation relative to other helices, in particular *α*-helices 7 and 10. Notably, the side chain of Lys338 is moved out of the hydrophobic pocket formed by Leu286, Tyr290, Tyr331, and Phe341. In the known crystal structure of the LBD of ESRRG, a similar pocket is contiguous with the second pocket of the LBD, which receives agonist ligand GSK4716 and coactivator RIP140 [14]. The ESRRG-RIP140 interaction is facilitated by conformational changes in the charged residues on the LBD surface. The hydrophobic pocket which includes Lys338 of ESRRB may have a similar function of allowing agonist ligand and coactivator binding by increasing the volume for ligand interaction and providing charged residues that are required for ligand binding. 

Three of five reported *ESRRB *mutations that cause ARNSHI are also predicted to result in structural defects at the LBD [1]. In particular, two *ESRRB *mutations, namely, p.Val342Leu and p.Leu347Pro [2], also occur within *α*-helix 8. Both substitutions are predicted to disrupt hydrophobic interactions of *α*-helix 8 with other helices, thus resulting in conformational change and decreased stability of the LBD [2]. Mutations in *α*-helix 8 that decrease ligand-binding affinity have been identified in other nuclear receptor proteins, ESR1 and HNF4*α*, which have structures similar to ESRRB [16, 17]. The p.Glu276Gln mutation for HNF4*α*, which is one of the underlying causes of mature-onset diabetes in the young, results in unstable protein with no DNA-binding ability and, hence, no transcriptional activity [16]. The Glu276 residue of HNF4*α* aligns with Glu340 of ESRRB. It is, therefore, highly plausible that p.Glu340del affects the stability and ligand-binding activity of the ESRRB protein in order to result in the HI phenotype.

## 4. Conclusion

A genome-wide linkage scan performed using DNA samples from a large consanguineous ARNSHI pedigree and the subsequent sequencing of the *ESRRB* gene led to the identification of a novel deletion mutation c.1018_1020delGAG (p.Glu340del). The glutamic acid residue 340 is located in the ligand-binding domain of the ESRRB protein. The identification of the deletion mutation of *ESRRB* gene will expand our understanding of hearing impairment due to mutations in the estrogen-related gene *ESRRB*.

## Figures and Tables

**Table 1:** Two-point and multipoint LOD scores for family 4243.

Marker name^1^	Physical map position^2^	Genetic map position^3^	Multipoint LOD score	Two-point LOD score at *θ* =					
				0.00	0.01	0.05	0.10	0.20	0.30
rs221924	70,650,531	66.96	−4.52	−2.02	−0.26	0.28	0.39	0.33	0.20
**rs917284**	**71,806,864 **	**67.80 **	−**2.56**	−1.41	0.34	0.84	0.90	0.70	0.36
rs1468507	73,641,851	70.09	4.15	2.67	2.62	2.40	2.12	1.54	0.94
rs935340	75,660,959	72.79	4.16	3.04	2.98	2.72	2.40	1.73	1.06
rs888412	77,676,577	75.73	4.16	1.34	1.30	1.13	0.91	0.46	0.02
**rs2043585**	**79,039,998 **	**77.88 **	−**17.85**	−∞	0.58	1.08	1.13	0.92	0.56
rs1530771	80,095,275	78.71	−∞	−∞	0.47	0.98	1.04	0.84	0.50
rs799032	81,707,098	79.67	−∞	−∞	0.84	1.32	1.35	1.09	0.69
rs1964413	82,338,459	80.75	−1.27	2.08	2.04	1.88	1.66	1.22	0.76
rs1885316	84,924,908	82.25	−6.89	−2.37	−0.60	−0.03	0.12	0.15	0.09
rs1956411	88,204,113	85.49	−5.94	−2.10	−0.33	0.22	0.36	0.34	0.22

^1^Markers in bold denote marker limits based on 3-unit support interval and homozygous region.

^2^Physical map positions from Build 36.1 of human reference sequence.

^3^Genetic map positions based on Rutgers linkage physical map of the human genome.
